# KUS121, a VCP modulator, attenuates ischemic retinal cell death via suppressing endoplasmic reticulum stress

**DOI:** 10.1038/srep44873

**Published:** 2017-03-20

**Authors:** Masayuki Hata, Hanako O. Ikeda, Chinami Kikkawa, Sachiko Iwai, Yuki Muraoka, Tomoko Hasegawa, Akira Kakizuka, Nagahisa Yoshimura

**Affiliations:** 1 Department of Ophthalmology and Visual Sciences, Kyoto University Graduate School of Medicine, Kyoto, Japan; 2 Neuroprotective Treatment Project for Ocular Diseases, Institute for Advancement of Clinical and Translational Science, Kyoto University Hospital, Kyoto, Japan; 3 Pharmacology Group, Non-clinical Research, Global R&D, Santen Pharmaceutical Co., Ltd, Nara, Japan; 4 Laboratory of Functional Biology, Kyoto University Graduate School of Biostudies & Solution Oriented Research for Science and Technology, Kyoto, Japan

## Abstract

Ischemic neural damages cause several devastating diseases, including brain stroke and ischemic retinopathies, and endoplasmic reticulum (ER) stress has been proposed to be the underlying mechanism of the neuronal cell death of these conditions. We previously synthesized Kyoto University substances (KUSs) as modulators of valosin-containing protein (VCP); KUSs inhibit VCP ATPase activity and protect cells from different cell death-inducing insults. Here, we examined the efficacy of KUS121 in a rat model of retinal ischemic injury. Systemic administration of KUS121 to rats with ischemic retinal injury significantly suppressed inner retinal thinning and death of retinal ganglion and amacrine cells, with a significant functional maintenance of visual functions, as judged by electroretinography. Furthermore, intravitreal injection of KUS121, which is the clinically preferred route of drug administration for retinal diseases, appeared to show an equal or better neuroprotective efficacy in the ischemic retina compared with systemic administration. Indeed, induction of the ER stress marker C/EBP homologous protein (CHOP) after the ischemic insult was significantly suppressed by KUS121 administration. Our study suggests VCP modulation by KUS as a promising novel therapeutic strategy for ischemic neuronal diseases.

Ischemic neural damages cause various devastating diseases, including brain stroke, a major fatal disease as well as the main cause of long-lasting disability^1^. The current treatment for brain stroke is largely limited to thrombolysis with tissue plasminogen activator, but only about 5% of stroke patients have the opportunity to receive this thrombolysis treatment^2^. Retinal ischemia has been proposed to contribute to the pathologies of several retinal diseases, including diabetic retinopathy, optic neuropathy, glaucoma, and retinal artery occlusion, and is an important cause of blindness^3^,^4^,^5^. No treatment effectively protects neurons from the ischemia of such diseases. Irreversible and progressive neuronal cell death, probably apoptosis, occurs following ischemia, and involvement of endoplasmic reticulum (ER) stress has been proposed as the underlying mechanism of the neuronal cell death^6^,^7^. Neuronal cells have a highly developed ER. Ischemia is an ER stressor that induces cell death in neural cells^8^,^9^,^10^. In particular, C/EBP homologous protein (CHOP), an ER stress-induced molecule, has been reported to be a major mediator of apoptosis in neural ischemia^7^. Therefore, new drugs or compounds with ER stress-reducing activities may have neuroprotective functions in ischemic diseases and investigation of their ability to ameliorate ischemic neural injury would be worthwhile.

Valosin-containing protein (VCP) is an AAA (ATPases Associated with diverse cellular Activities)-type ATPase with a ubiquitous expression that has been proposed to be a major player in neurodegeneration^11^,^12^,^13^. In addition to its ATPase activity, there are many proposed cellular functions of VCP, such as proteasome-mediated protein degradation and endoplasmic reticulum-associated degradation^14^,^15^,^16^. We previously showed that KUSs (Kyoto University Substances), new compounds developed as ATPase inhibitors of VCP, have novel functions as “VCP modulators” or “ATP regulators” and specifically inhibit the ATPase activities of VCP without apparent inhibition of its cellular functions^17^. These “VCP modulators” have strong neuroprotective effects *in vivo* on retinal photoreceptor cells and ganglion cells^17^,^18^,^19^. These efficacies apparently correlate with their ability to ameliorate ER stress. Given that the major pathology of ischemic diseases is also ER stress-induced cell death, KUSs may provide a novel strategy for cell protection in such incurable disorders. Hence, we investigated the effect of a KUS on the retina after ischemic injury by analyzing the morphology, retinal function, and extents of ER stress and cell death.

## Results

**Figure 1:**
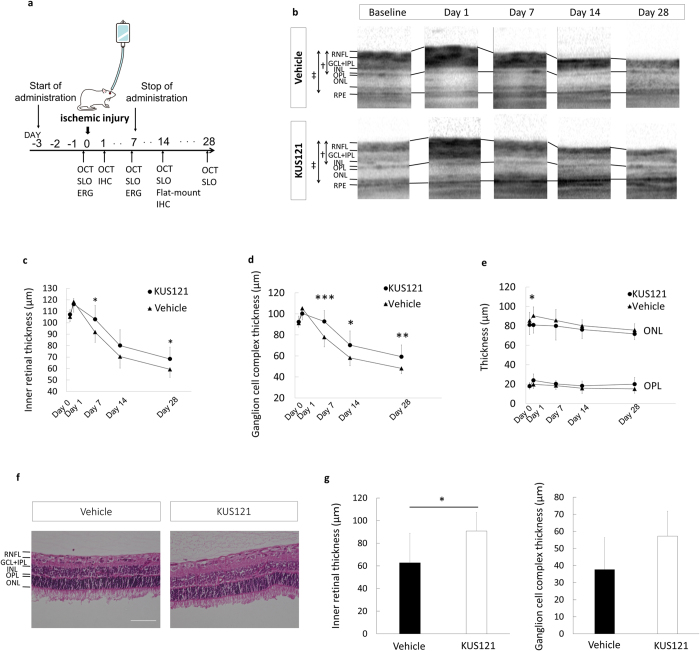
Effects of VCP modulators on inner retinal thickness in an ischemic retinal injury model. (**a**) Experimental system of the retinal ischemic rat model. (**b–e**) Longitudinal evaluation of retinal thickness by SD-OCT in rats subjected to ischemia treated with KUS121 or PBS (control). (**b**) Representative SD-OCT images. ^†^Inner retinal thickness, ^‡^total retinal thickness. (**c**) Inner retinal thickness in control (*n* = 12, *triangles*) and KUS121-treated (*n* = 12, *circles*) groups. Error bars indicate SD. *P < 0.05 vs. control (Student’s *t* test). (**d**) Ganglion cell complex thickness in control (*n* = 12, *triangles*) and KUS121-treated (*n* = 12, *circles*) groups. Error bars indicate SD. *P < 0.05, **P < 0.01 and ***P < 0.005 vs. control (Student’s *t*-test). (**e**) Outer plexiform layer (OPL) thickness and outer nuclear layer (ONL) thickness in control (*n* = 12, *triangles*) and KUS121-treated (*n* = 12, *circles*) groups. Error bars indicate SD. *P < 0.05 vs. control (Student’s *t*-test). (**f**,**g**) Evaluation of retinal thickness of hematoxylin & eosin (HE)-stained retinal sections of rats subjected to ischemia and treated with KUS121 or PBS (control). (**f**) Representative images of HE-stained retinal sections. Bar = 50 μm. (**g**) Inner retinal thickness and ganglion cell complex thickness in control and KUS121-treated rats (*n* = 8 per group). Error bars indicate SD. *P < 0.05 vs. control (Student’s *t*-test).

To investigate whether the VCP modulator possesses neuroprotective effects against ischemic neural cell damage, we employed a rat model of ischemic retinal injury. In this model, the intraocular pressures of *Thy1*-green fluorescent protein (GFP) rats were raised to 120 mm Hg for 60 min to induce retinal ischemia (Fig. 1a, see Methods).

First, we examined the time-dependent changes in retinal thickness in the eyes of *Thy1*-GFP rats with ischemic retinal injury by using spectral domain-optical coherent tomography (SD-OCT) images to detect the retinal damage or atrophy caused by ischemic retinal injury. In both groups intraperitoneally treated with KUS121 or phosphate-buffered saline (PBS; control group), total retinal thickness increased at day 1 and decreased thereafter (Fig. 1b). The inner retina [composed of the retinal nerve fiber layer, retinal ganglion cells (RGCs), inner plexiform layer, and inner nuclear layer], which is located under the retinal vessel supply, was primarily impaired by the ischemic insult. In particular, the ganglion cell complex (composed of the retinal nerve fiber layer, RGCs, and inner plexiform layer) was the structure damaged by the ischemic insult (Fig. 1b). On the other hand, the outer retina was less affected by ischemic insult. Importantly, compared with the control group, the KUS121-treated group showed a significant suppression of inner retinal thinning at days 7 (103.1 ± 11.9 μm vs. 91.8 ± 9.0 μm, mean ± standard deviation, *P* = 0.021) and 28 (68.4 ± 10.3 μm vs. 59.3 ± 7.0 μm, *P* = 0.019; Fig. 1c). The ganglion cell complex was also significantly protected from damage at days 7 (92.7 ± 10.3 μm vs. 77.9 ± 9.3 μm, *P* = 0.002), 14 (70.3 ± 13.5 μm vs. 58.2 ± 7.4 μm, *P* = 0.014), and 28 (59.3 ± 11.1 μm vs. 48.2 ± 5.1 μm, *P* = 0.005; Fig. 1d). In the outer retina, no differences in thickness of the outer nuclear layer and outer plexiform layer were observed between KUS121-treated and control groups, except for outer nuclear layer thickness on day 1 (*P* = 0.019, Fig. 1e). Hematoxylin & eosin staining of retinal sections at day 14 after the retinal ischemic injury also revealed significant suppression of inner retinal thinning in KUS121-treated rats (*P* = 0.041, Fig. 1f,g).

**Figure 2:**
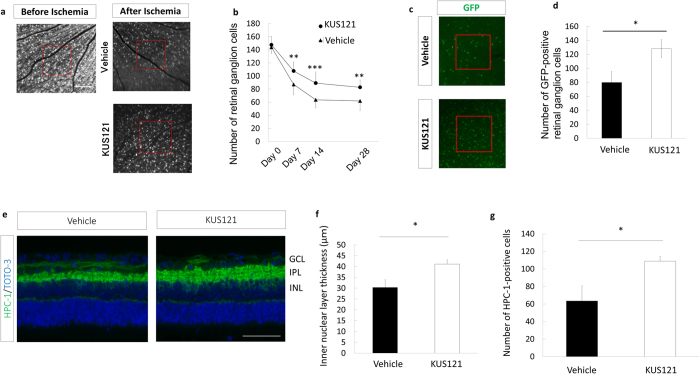
Effects of VCP modulators on retinal cells in an ischemic retinal injury model. (**a**,**b**) RGC numbers in the ischemic retina using SLO imaging. (**a**) Representative images from animals treated with KUS121 or not 28 days after ischemia. Bar = 500 μm. (**b**) Longitudinal changes in RGCs counted within the red boxes in **a** in control (*n* = 11, *triangles*) and KUS121-treated (*n* = 11, *circles*) groups. Error bars indicate SD. **P < 0.01 and ***P < 0.005 vs. control (Student’s *t* test). (**c** and **d**) GFP-positive RGC numbers in flat-mounted retinas treated with KUS121 or not 14 days after ischemia. (**c**) Representative images. (**d**) GFP-positive RGC numbers counted within the red boxes in **c** in control (*n* = 8) and KUS121-treated (*n* = 10) groups. Error bars indicate SE. *P < 0.05 vs. control (Student’s *t* test). (**e**–**g**) Retinal section analysis 14 days after ischemic retinal injury. (**e**) Immunohistochemical images of retinal sections stained with HPC-1 and TOTO-3. Bar = 50 μm. (**f**) Inner nuclear layer thickness (each group, *n* = 8). *P < 0.05 vs. control (Student’s *t* test). (**g**) Numbers of HPC-1–positive cells in the inner nuclear layer (corresponding to amacrine cells) (each group, *n* = 8). *P < 0.05 vs. control (Aspin-Welch *t* test).

To investigate the neuroprotective effects of KUS121 on the RGC layer, we used scanning laser ophthalmoscope (SLO) to examine the time-dependent changes in the numbers of RGCs remaining after the ischemic insult. While the average number of the remaining RGCs detected after the ischemic insult decreased in both groups (Fig. 2a,b, and Supplemental Fig. 1a), the number of remaining RGCs was significantly preserved in the rats treated with KUS121 (day 7, 107.7 ± 17.3 vs. 87.2 ± 13.2, *P* = 0.005; day 14, 89.3 ± 16.5 vs. 63.8 ± 14.1, *P* = 0.001; day 28, 82.9 ± 20.2 vs. 62.0 ± 13.9, *P* = 0.009; Fig. 2a,b). This neuroprotective effect on the RGC layer was also confirmed by assessing the GFP signal in whole-mounted retina 14 days after the ischemic insult (128.2 ± 12.9 vs. 79.9 ± 15.6, *P* = 0.037; Fig. 2c,d and Supplemental Fig. 1b). In addition to RGC survival, to determine the neuroprotective effect of KUS121 on amacrine cells, we performed immunohistochemical analyses by using an anti–HPC-1 antibody to detect amacrine cells remaining in paraffin-embedded sections. Histological analyses revealed that inner nuclear layer thickness and amacrine cell numbers were significantly preserved in KUS121-treated rats at 14 days after the ischemic insult (inner nuclear layer thickness, 41.1 ± 2.0 μm vs. 30.4 ± 3.6 μm, *P* = 0.028; amacrine cell number, 108.9 ± 5.6 vs. 63.5 ± 17.3, *P* = 0.035; Fig. 2e–g).

**Figure 3:**
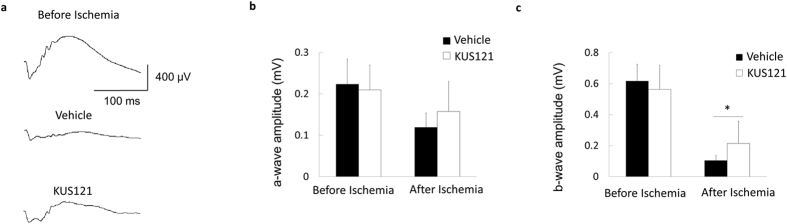
Effects of VCP modulators on retinal function in an ischemic retinal injury model. (**a**–**c**) Scotopic electroretinography with intensities of 3 cd·s·m^−2^ before and 7 days after ischemia. (**a**) Representative images. (**b**) a-wave amplitudes (each group, *n* = 12). Error bars indicate SD. (**c**) b-wave amplitudes (each group, *n* = 12). Error bars indicate SD. *P < 0.05 vs. control (Aspin-Welch *t* test).

We performed electroretinography (ERG) and assessed b-wave amplitudes to confirm whether the neuroprotective effects of KUS121 are mirrored by preservation of visual functions after ischemic retinal injury. Indeed, KUS121 preserved b-wave amplitude compared with that of the vehicle group 2 weeks after the ischemic insult (213.8 ± 143.5 μV vs. 103.8 ± 34.2 μV, *P* = 0.040; Fig. 3a,c). The KUS121-treated and control groups showed marginally significant differences in terms of a-wave amplitude (157.6 ± 72.5 μV vs. 119.3 ± 34.4 μV, *P* = 0.170; Fig. 3b). Retinal ischemia caused delay of a- and b- wave latencies (*P* = 0.001, *P* = 0.002). However, no significant differences in a-wave or b-wave latencies were observed between KUS121-treated and control rats (*P* = 0.194, *P* = 0.416).

**Figure 4:**
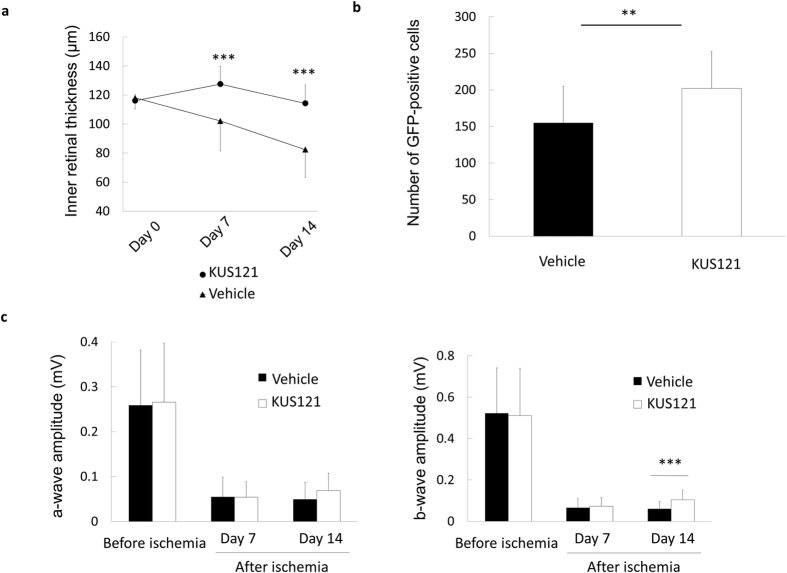
Effects of intravitreal injections of VCP modulator on retinal ischemic rat model. (**a**) Longitudinal changes in inner retinal thickness in the control (*n* = 13, *triangles*) and IVT-KUS121–treated (*n* = 11, *circles*) groups. Inner retinal thickness was significantly greater in the IVT-KUS121–treated group than in the control group 7 and 14 days after ischemic retinal injury. Error bars indicate SD. ***P < 0.001 vs. control (Student’s *t* test). (**b**) RGC number in the control (*n* = 15) and IVT-KUS121–treated (*n* = 20) groups, confirming significant preservation of RGC number in the IVT-KUS121–treated group compared with the control group after ischemic retinal injury. Error bars indicate SE. **P < 0.01 vs. control (Student’s *t* test). (**c**) a-wave and b-wave amplitudes of electroretinography recordings of rats in the control (*n* = 21) and IVT-KUS121–treated (*n* = 24) groups before ischemic injury, 7 days after ischemic injury, and 14 days after ischemic injury. Rats treated with IVT-KUS121 showed a significant preservation of b-wave amplitude 14 days after ischemic injury. Error bars indicate SD. ***P < 0.005 vs. control (Aspin-Welch *t* test).

We evaluated the efficacy of intravitreal (IVT) injection of KUS121 in the ischemic retinal injury model, considering that IVT is the clinically preferred route of drug administration for retinal diseases. Compared with systemic administration of KUS121, IVT injections of KUS121 appeared to show a better suppression of inner retinal thinning 7 and 14 days after the ischemic insult (day 7, 127.6 ± 12.0 μm vs. 102.2 ± 20.6 μm, *P* = 0.0008; day 14, 114.3 ± 12.6 μm vs. 82.4 ± 19.4 μm, *P* = 0.0001; Fig. 4a). IVT injections of KUS121 also showed a better preservation of GFP-positive RGCs and equally preserved b-wave amplitude 14 days after the insult (RGC number, 202.2 ± 47.9 vs. 154.8 ± 50.5, *P* = 0.0078; b-wave amplitude, 104.5 ± 48.1 μV vs. 61.3 ± 35.6 μV, *P* = 0.002; Fig. 4b,c). A marginally significant difference in a-wave amplitude was observed between KUS121-treated and control groups 14 days following ischemic insult (68.8 ± 38.7 μV vs. 49.7 ± 37.7 μV, *P* = 0.108; Fig. 4c). There were no significant differences in a-wave or b-wave latencies between KUS121-treated and control groups (a-wave, *P* = 0.067 at day 7, *P* = 0.258 at day 14; b-wave, *P* = 0.957 at day 7, *P* = 0.395 at day 14).

**Figure 5:**
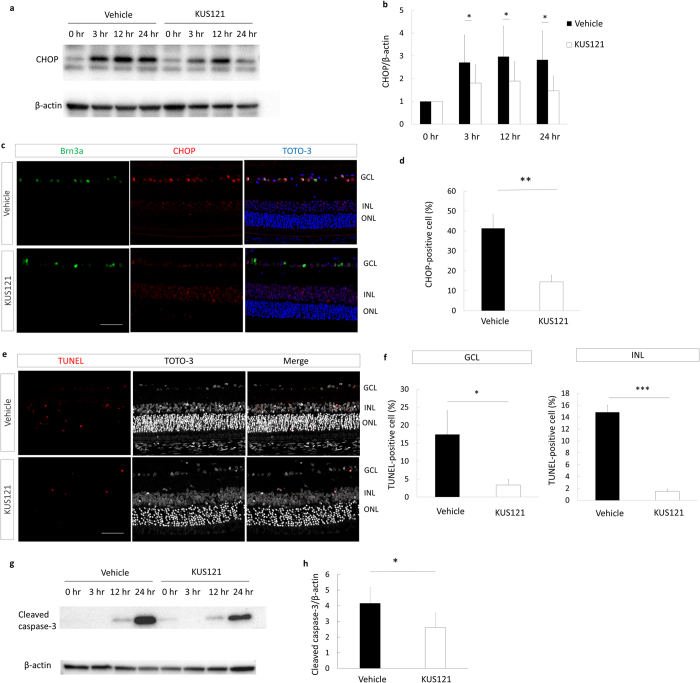
Mechanisms of the neuroprotective effects of VCP modulator on neural ischemia. (**a**) Western blot analysis of the CHOP and beta-actin expression of rat retina before ischemic injury and 3, 12, and 24 h after ischemic injury. Ischemic injury increased the expression level of CHOP in the retina. (**b**) Statistical analysis of the CHOP and beta-actin expression of rat retina after ischemic retinal injury (each point of each group, *n* = 5), indicating that the protein level of CHOP was significantly lower in the KUS121 group than in the control group 3, 12, and 24 h after ischemic retinal injury. Error bars indicate SD. *P < 0.05 vs. control (Aspin-Welch *t* test). (**c**) Immunohistochemical images of retinal sections stained with Brn3a, CHOP, and TOTO-3 24 h after ischemic retinal injury, showing the lower percentage of CHOP-positive RGCs in the KUS121-treated rat than in the vehicle-treated rat. Bar = 50 μm. (**d**) Numbers of CHOP-positive cells in the ischemic retina, showing that KUS121 significantly decreased ER stress in the RGCs (each group, *n* = 11). **P < 0.01 vs. control (Aspin-Welch *t* test). (**e**) Representative TUNEL staining of retinal sections 24 h after ischemic retinal injury. There are fewer TUNEL-positive cells in the ganglion cell layer (GCL) and inner nuclear layers (INL) in the KUS121-treated group than in the control group. Bar = 50 μm. (**f**) Analysis of the number of apoptotic cells within four 780-μm squares at a distance of 500 μm from the center of the optic nerve head, showing that KUS121 significantly decreased cell apoptosis in the GCL and INL (each group, *n* = 8). **P < 0.05 and ***P < 0.005 vs. control (Aspin-Welch *t* test). (**g**) Western blot analysis of cleaved caspase-3 and beta-actin in the rat retina before ischemic injury and 3, 12, and 24 h after ischemic injury. Ischemic injury resulted in cleaved caspase-3 upregulation. (**h**) The protein levels of cleaved caspase-3 were significantly lower in the KUS121 group than in the control group at 24 h after ischemic retinal injury (each group, *n* = 5). Error bars indicate SD. *P < 0.05 vs. control (Aspin-Welch *t* test).

To examine the mechanisms underlie the pathological and KUS treatment processes of ischemic retinal injury, we examined ER stress and apoptosis markers after the ischemic insult. CHOP protein was clearly induced as early as 3 hours after the ischemic insult and its expression was retained up to 24 hours after the insult (Fig. 5a). Strikingly, in the ischemic retinal-injured rats treated with KUS121, the protein levels of CHOP were significantly decreased compared with the non-KUS–treated control group (control group) 3, 12, and 24 hours after the ischemic insult (*P* = 0.018, *P* = 0.044, and *P* = 0.011, respectively; Fig. 5b). Immunohistochemical analyses of retinal sections showed that fewer CHOP-positive RGCs were observed in the KUS121-treated group than in the control group (41.4 ± 7.0% vs. 14.5 ± 3.3%, *P* = 0.005; Fig. 5c,d). In addition, there were significantly fewer TdT-mediated dUTP Nick-end Labeling (TUNEL)-positive apoptotic cells in the ganglion cell layer (GCL) and inner nuclear layers of the KUS121-treated group than in the control group (GCL, 17.4 ± 6.6% vs. 3.4 ± 1.5%, *P* = 0.038; inner nuclear layer, 14.8 ± 1.2% vs. 1.5 ± 0.4%, *P* = 0.001; Fig. 5e,f). Cleaved caspase-3 was upregulated 24 h following ischemic insult in the control group (Fig. 5g). On the other hand, expression of cleaved caspase-3 in KUS121-treated retinas was significantly downregulated compared to that in the vehicle-treated control group (*P* = 0.032; Fig. 5g,h).

## Discussion

In the present study, we examined the efficacy of KUS121 in a rat model of retinal ischemic injury. Systemic administration of KUS121 into rats subjected to ischemic retinal injury significantly suppressed inner retinal thinning and apoptosis of retinal ganglion and amacrine cells, with a significant functional preservation of visual function by suppressing ER stress. Furthermore, intravitreal injection of KUS121 appeared to exhibit equal or better neuroprotective efficacy in the ischemic retina compared to systemic administration.

In our previous studies, KUS121 showed significant neuroprotective effects in rd10 mice, rd12 mice, and mutated rhodopsin transgenic rabbits, which are models of retinitis pigmentosa^17^,^19^. Similar results were obtained using three different mouse models of glaucoma but without observable side effects^18^. KUSs exhibited ER stress-reducing effects, demonstrating the neuroprotective effects on retinal cells in these mouse models. The pathology of ischemic neural cell death has been proposed to involve apoptotic cell death caused by ER stress^6^,^7^. We thus evaluated the effect of KUS121 on the retinal ischemic injury model. Results showed that KUS121 also exhibited potent anti-apoptotic activity in retinal cells under ischemic conditions. In addition, ER stress in retinal cells was consistently suppressed by KUS in the ischemic retinal injury model, as well as in retinitis pigmentosa and glaucoma models^17^,^18^,^19^, and thus represents the mechanism most likely to be responsible for the neuroprotective effects of KUSs.

*Thy1*-GFP transgenic rats expressing GFP in RGCs^20^ were used to non-invasively examine longitudinal changes in the retinal layer structure and determine RGC survival. We observed longitudinal thinning of the inner retina and a decrease in the number of surviving RGCs after the ischemic insult, consistent with results from previous studies on animal models and human subjects subjected to ischemic retinal injury^21^,^22^. By contrast, the outer retina, which is located under the choroidal vessel supply, showed less pronounced structural damage as a result of the ischemic insult.

We demonstrated that KUS121 could preserve retinal morphologies by protecting RGCs and amacrine cells from cell death after an ischemic insult. First, we examined the effect of KUS121 on longitudinal retinal thinning and demonstrated that KUS121 treatment significantly suppressed inner retinal thinning upon ischemic insult. These results suggest that KUS121 exerts a protective effect on RGCs and amacrine cells, cells of the inner retina that have been reported to be most vulnerable to ischemic stress^23^. To further investigate the neuroprotective effects of KUS121 on the RGC layer, we examined the longitudinal changes in the numbers of remaining RGCs following ischemic insult. Consistent with histological analyses, the number of remaining RGCs was significantly preserved in rats treated with KUS121. In addition to RGC survival, histological analyses revealed that KUS121 treatment significantly increased amacrine cell survival when subjected to ischemic insult.

KUS121 exerts not only morphological but also functional neuroprotective effects on the inner retina during retinal ischemia. Retinal function can be non-invasively and objectively measured using ERG. Ischemic retinal injury is observed to cause retinal dysfunction based on decreased b-wave amplitude, which reflects the responses of amacrine, Müller, and bipolar cells in ERG^24^,^25^,^26^ and suggests that the b-wave can be used as an indicator of ischemic injury severity^27^. In the present study, we noted dramatic reductions in b-wave amplitude but significant reductions in both a-wave amplitude and b-wave after the ischemic insult. These results are consistent with a previous study demonstrating the discrepancies between morphological and functional changes in the outer retina when subjected to ischemic retinal injury^23^. Furthermore, KUS121 treatment resulted in better preservation of b-wave amplitude than that seen in the vehicle group after ischemic insult; however, KUS121 showed limited effects on a-wave amplitude. Our results suggest that KUS121 exerts functional neuroprotective effects mainly on the inner retina, the structure primarily damaged during retinal ischemia.

In the present study, IVT injections of KUS121 also exerted neuroprotective effects on retinal morphology and function after ischemic damage. IVT injection is the drug delivery route more preferred than systemic administration for the treatment of retinal diseases. IVT injection enables faster delivery of effective amounts of a drug to the retina, especially under conditions of disturbed retinal blood supply, which typically occurs in retinal ischemic diseases. IVT injection is also suitable for lowering drug dosage, which in turn reduces the risk of side effects. Indeed, this approach has been effective in the treatment of various conditions, including age-related macular degeneration, viral retinitis, uveitis, and diabetic retinopathy^28^,^29^,^30^,^31^. Our study demonstrated that IVT injection of KUS121 was more effective than systemic administration in preserving retinal morphology and function after ischemic insult, thereby demonstrating the potential of KUS121 for future clinical trials in the treatment of ischemic retinal diseases.

In summary, we showed that the VCP modulator KUS121 exerts a profound anti-apoptotic effect on ischemic retinal injury by suppressing ER stress, resulting in morphological and functional neuroprotection. Our findings may provide a novel therapeutic strategy for the treatment of neural ischemic diseases.

## Methods

### Experimental animals

All studies were conducted in compliance with the ARVO Statement for the Use of Animals in Ophthalmic and Vision Research. All protocols were approved by the Institutional Review Board of the Kyoto University Graduate School of Medicine (MedKyo 14213, 15531, 16501). We used *Thy1*- GFP transgenic rats, which express GFP in ganglion cells, including RGCs, under the control of neuron-specific elements from the Thy1 gene (the transgenic Sprague Dawley rats were a gift of Dr. Christina K. Magill and Dr. Susan E. Mackinnon at Washington University)^15^. Animals were maintained under a 12-hour light:dark cycle. All rats were fed ad libitum. Male rats (age, 6–8 weeks; weight, 200–250 g) were used in the experiments. Before ischemia induction, image acquisition, ERG, and intravitreal injections, rats were anesthetized by an intraperitoneal injection of ketamine and xylazine (75 mg/kg and 5 mg/kg body weight, respectively). Pupils were dilated with tropicamide and phenylephrine eye drops (0.5% each).

### KUS treatment

For intraperitoneal injection, KUS121 was dissolved in 5% Cremophor EL (Sigma)/phosphate-buffered saline (PBS) to obtain a 5 mg/mL solution. For intravitreal injection, KUS121 was dissolved in PBS to obtain a 5 mg/mL solution. In the group treated with intraperitoneal injections, KUS121 (50 mg/kg/day) or 5% Cremophor/PBS was injected into the *Thy1*-GFP rats once daily between 3 days before and 7 days after the ischemic retinal injury (*n* = 59 and *n* = 61, respectively). Twelve rats of each group underwent spectral-domain optical coherence tomography (SD-OCT), scanning laser ophthalmoscope (SLO), and ERG analysis. Eight rats treated with vehicle and 10 rats treated with KUS121 were used for flat-mounted retinal imaging. Nineteen rats of each group were used for histological analyses. Five rats were used for western blotting analyses at each time point in each group. In the group treated with intravitreal injections, KUS121 (25 μg/eye) or PBS was injected into the vitreous of *Th1*-GFP rats using a 33-gauge needle (Ito Corporation) 2 hours before the ischemic retinal injury (*n* = 24 and *n* = 21, respectively).

### Retinal ischemic injury

Under anesthesia, the anterior chamber of the right eye in each rat was cannulated with a 33-gauge infusion needle connected to a reservoir containing normal saline. Intraocular pressure was raised to 120 mmHg for 60 min by elevating the saline reservoir. Only the right eye of each rat was subjected to ischemia. Retinal ischemia was confirmed by whitening of the fundus vessels. The left eye of each rat served as the nonischemic control.

### SD-OCT image acquisition and measurement of retinal thickness

SD-OCT examinations using Multiline OCT (based on a Spectralis HRA + OCT, Heidelberg Engineering)^26^ were performed in *Thy1*-GFP rats at 0, 1, 7, 14, and 28 days after the retinal ischemic injury. Inner retinal thickness (inner retina [IR] = retinal nerve fiber layer + ganglion cell layer + inner plexiform layer + inner nuclear layer), ganglion cell complex thickness (GCC, ganglion cell complex = retinal nerve fiber layer + ganglion cell layer + inner plexiform layer), outer nuclear layer thickness, and outer plexiform layer thickness were measured using circle scan images within a circle 0.944 mm in diameter, the center of which was adjusted to the center of the optic nerve head.

### Manual measurement of retinal layer thickness

The software used for drawing boundary lines was based on the built-in Spectralis HRA + OCT and provided by Heidelberg Engineering software to facilitate manual assessment of the B-scan images. This custom-made software allows various boundary lines to be drawn in each B-scan image. The software calculates the distance between the two manually drawn boundary lines for each layer of interest to yield a thickness value at each location. A boundary line was automatically placed along the border of the internal limiting membrane (ILM) and the vitreous, and other boundary lines were manually placed between the inner nuclear layer and outer plexiform layer, between the inner plexiform and inner nuclear layer, and between the outer plexiform and outer nuclear layer in a masked fashion. The distances between the ILM and the outer border of inner nuclear layer, the ILM and the outer border of inner plexiform layer, the inner border and the outer border of outer plexiform layer, and the inner border of outer nuclear layer and the inner border of retinal pigment epithelium were calculated as the IR thickness, GCC thickness, outer plexiform layer thickness, and outer nuclear layer thickness, respectively. The mean thickness in each circular scan was calculated by the software.

### Counting of RGCs in SLO images

GFP-positive RGCs were manually counted within four 780-μm squares at a distance of 1560 μm from the center of the optic nerve head on the SLO images in a masked fashion. We selected the best images and determined the best areas in which the cells in the GCL were sharply focused. The numbers of RGCs counted in the four square areas were averaged.

### RGC counting in flat-mounted retinal images

The number of GFP-positive cells in the retina was also assessed using whole-mounted retina. Fourteen days after the retinal ischemic injury, the eyeballs were removed and the retina was whole mounted and imaged with a fluorescence microscope (BZ9000; KEYENCE). GFP-positive RGCs were manually counted within four 500-μm squares at a distance of 2000 μm from the center of the optic nerve head on the flat-mounted retinal images in a masked fashion. Cell counting was performed by an experimental investigator (AH) who was masked to the experimental treatment of the eye. The numbers of RGCs counted in the four square areas were averaged.

### ERG

ERG recording was performed to assess the visual function of *Thy1*-GFP rats 7 days after the retinal ischemic injury. Rats were dark-adapted overnight before anesthetization. ERGs were recorded using a gold loop corneal electrode with a light-emitting diode (Mayo Corp.). A reference electrode was placed in the mouth and a ground electrode was attached to the tail. Stimuli were produced with a light-emitting diode stimulator (Mayo Corp.). Full-field ERGs were recorded with a Ganzfeld sphere and with single flashes with intensities of 3 cd·s·m^−2^. The electroretinogram response signals were amplified, digitized at 10 kHz with a band-pass filter of 0.3 to 500 Hz, and analyzed (PowerLab 2/25; AD Instruments). The a- and b-wave amplitudes of the mixed cone and rod response (ISCEV [International Society for Clinical Electrophysiology of Vision] standard; scotopic 3.0^27^) were analyzed.

### Antibodies

Anti-CHOP and anti-CHOP antibodies were purchased from Chemicon; anti-actin antibody from Millipore; anti-Brn3a antibody from Santa Cruz; and anti-HPC-1 antibody from Sigma-Aldrich; anti-cleaved caspase-3 antibody was purchased from Cell Signaling Technology.

### Histological analyses

For histological analysis, rats were anesthetized by the methods described at 1 day or 14 days after the retinal ischemic injury. The eyes were fixed in 4% paraformaldehyde for 24 hours at 4 °C and embedded in paraffin. Serial 6-mm paraffin-embedded sections passing through the center of the optic nerve head were selected. Hematoxylin & eosin staining was used to evaluate changes in the thickness of the inner retina. Immunohistochemical staining was performed with antibodies to CHOP, Brn3a (as a marker of RGC cells), and HPC-1 (as a marker of amacrine cells). Nuclei were counterstained with a red fluorescent dye (TOTO-3; Molecular Probes). The ratio of the number of cells positive for CHOP among the cells positive for Brn3a in the GCL in the whole vertical sections (through the optic nerve head) of the retinas collected 24 hours after the retinal ischemic injury (*n* = 11) was calculated on images acquired with fluorescence microscopy (BZ9000; KEYENCE). The ratio of the number of HPC1-positive cells in the INL in the whole vertical sections (through the optic nerve head) of the retinas collected 14 days after the retinal ischemic injury (*n* = 8) was calculated on images acquired with the BZ9000 fluorescence microscope. The inner retinal thickness was also measured on the same sections.

### Western blotting

Eyeballs were immediately immersed in cold Hanks’ balanced salt solution after enucleation. Incisions were made by making pinholes in the corneas, after which the sclera, choroid, and retinal pigment epithelium were peeled from the incisions to collect the neural retina. The lens and iris were removed. Five retinas at each time point (before or 3, 12, and 24 h after ischemic injury) were independently studied.

### *In situ* TdT-mediated dUTP Nick-end Labeling (TUNEL) Assay

Retinal cell apoptosis was determined by a TUNEL assay 24 hours after the retinal ischemic injury. The retinal sections were stained using a TUNEL-based kit (FragEL^TM^ DNA Fragmentation Detection Kit, Colorimetric-TdT Enzyme; Calbiochem) according to the manufacturer’s instructions. The ratio of the number of TUNEL-positive cells in the GCL and INL in the whole vertical sections (through the optic nerve head) of the retinas collected 24 hours after the retinal ischemic injury (*n* = 8) was calculated on images acquired using a BZ9000 fluorescence microscope.

### Statistics

Variables were compared among rats treated with or without VCP modulators with the Aspin-Welch *t* test or Student’s *t* test. Statistical analyses were performed using SPSS Statistics version 17.0 (SPSS Inc.). The level of statistical significance was set at *P* < 0.05.

### Study approval

All studies were conducted in compliance with the ARVO Statement for the Use of Animals in Ophthalmic and Vision Research. All protocols were approved by the Institutional Review Board of Kyoto University Graduate School of Medicine (MedKyo14213, 15531, 16501).

## Additional Information

**How to cite this article:** Hata, M. *et al*. KUS121, a VCP modulator, attenuates ischemic retinal cell death via suppressing endoplasmic reticulum stress. *Sci. Rep.* **7**, 44873; doi: 10.1038/srep44873 (2017).

**Publisher's note:** Springer Nature remains neutral with regard to jurisdictional claims in published maps and institutional affiliations.

## Supplementary Material

Supplemental Figure 1
