# Supplementary material for: KUS121, a VCP modulator, attenuates ischemic retinal cell death via suppressing endoplasmic reticulum stress
Source: Sci Rep. 2017 Mar 20;7:44873. doi: 10.1038/srep44873 (PMC5357950; doi:10.1038/srep44873)

Title: KUS121, a VCP modulator, attenuates ischemic retinal cell death via suppressing endoplasmic reticulum stress

Masayuki Hata, Hanako O. Ikeda, Chinami Kikkawa, Sachiko Iwai, Yuki Muraoka, Tomoko Hasegawa, Akira Kakizuka, and Nagahisa Yoshimura

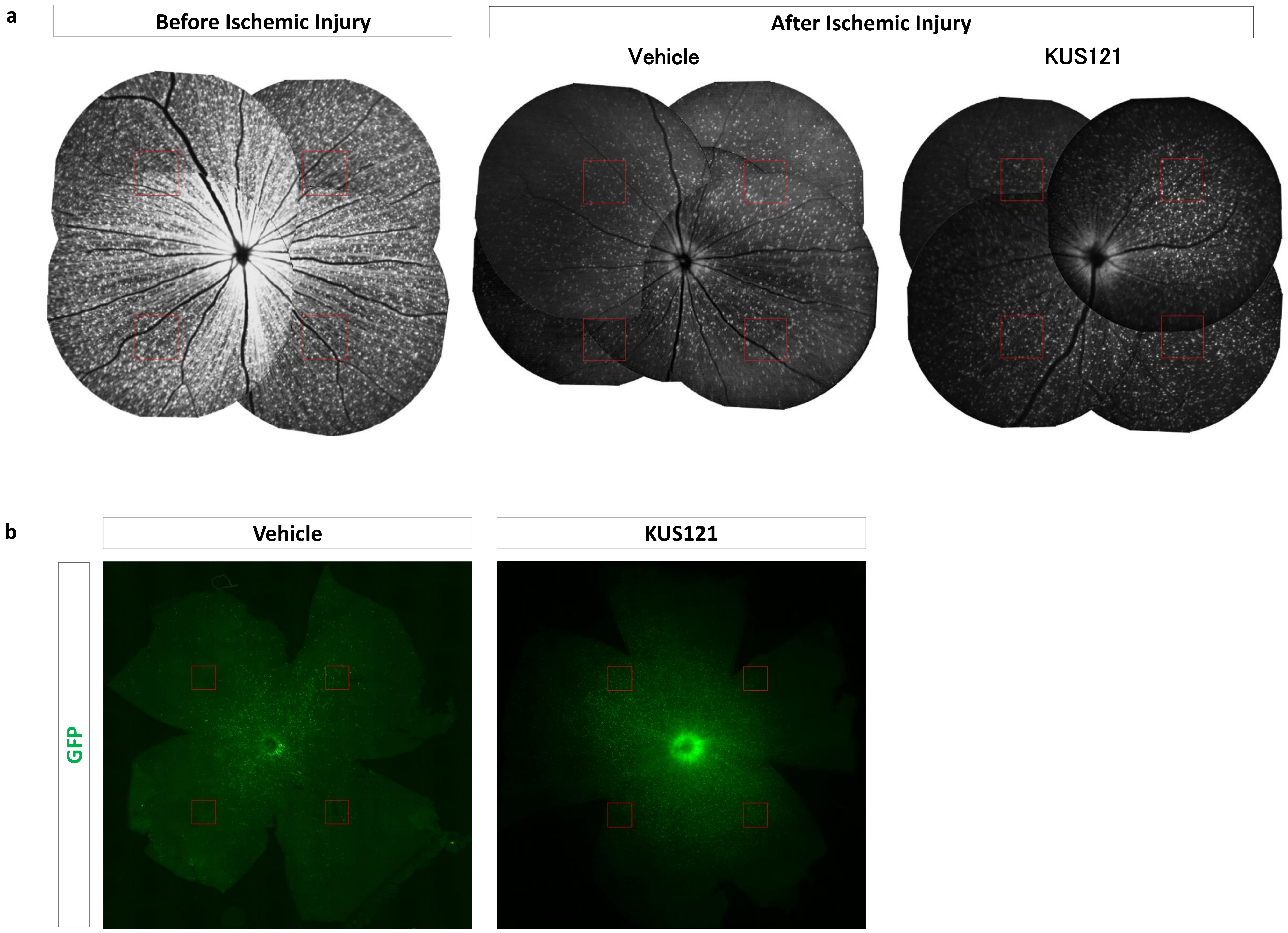

Supplemental Figure 1. Low-magnification images of SLO imaging and flat-mounted retinas of rats. (**a**) Low-magnification images of RGCs in rats before ischemia and those treated with KUS121 or not 28 days after ischemia using SLO imaging. (**b**) Low-magnification images of flat-mounted retinas of rats treated with KUS121 or not 14 days after ischemic retinal injury.

Full unedited gel for Figure 5a

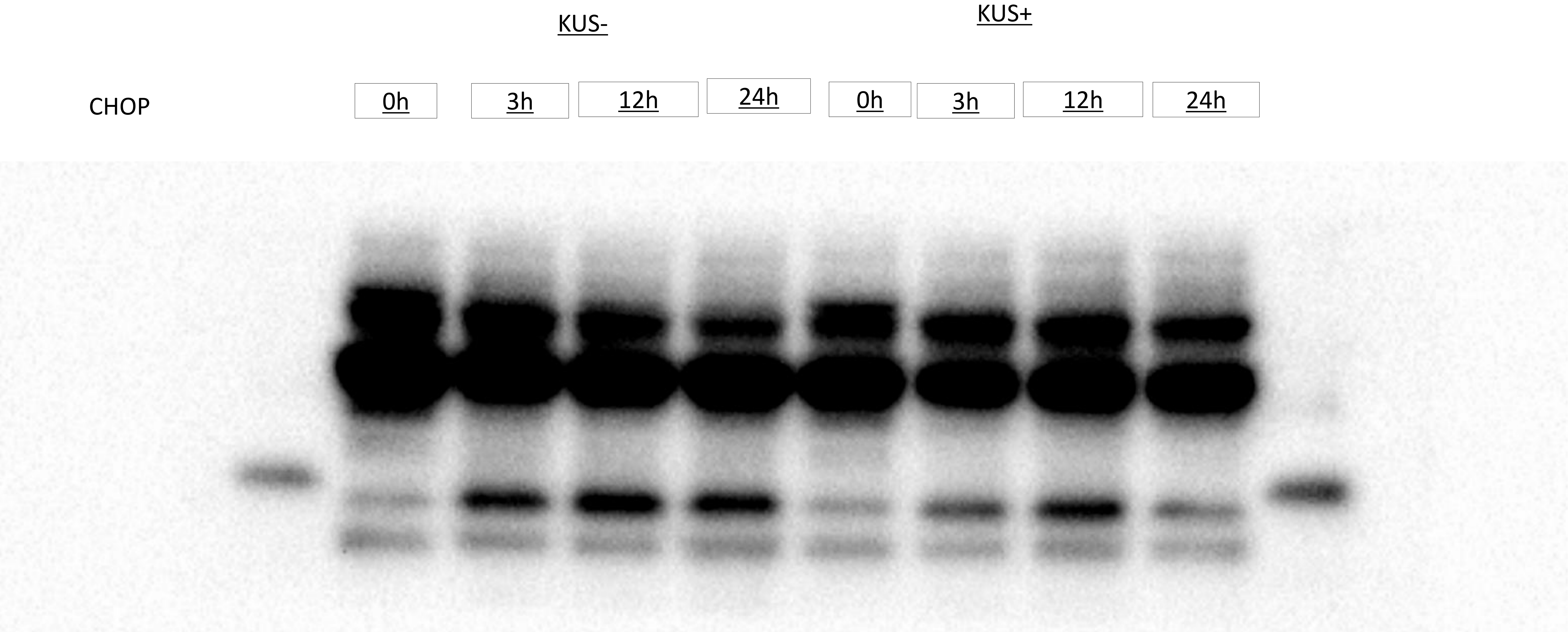

Full unedited gel for Figure 5a

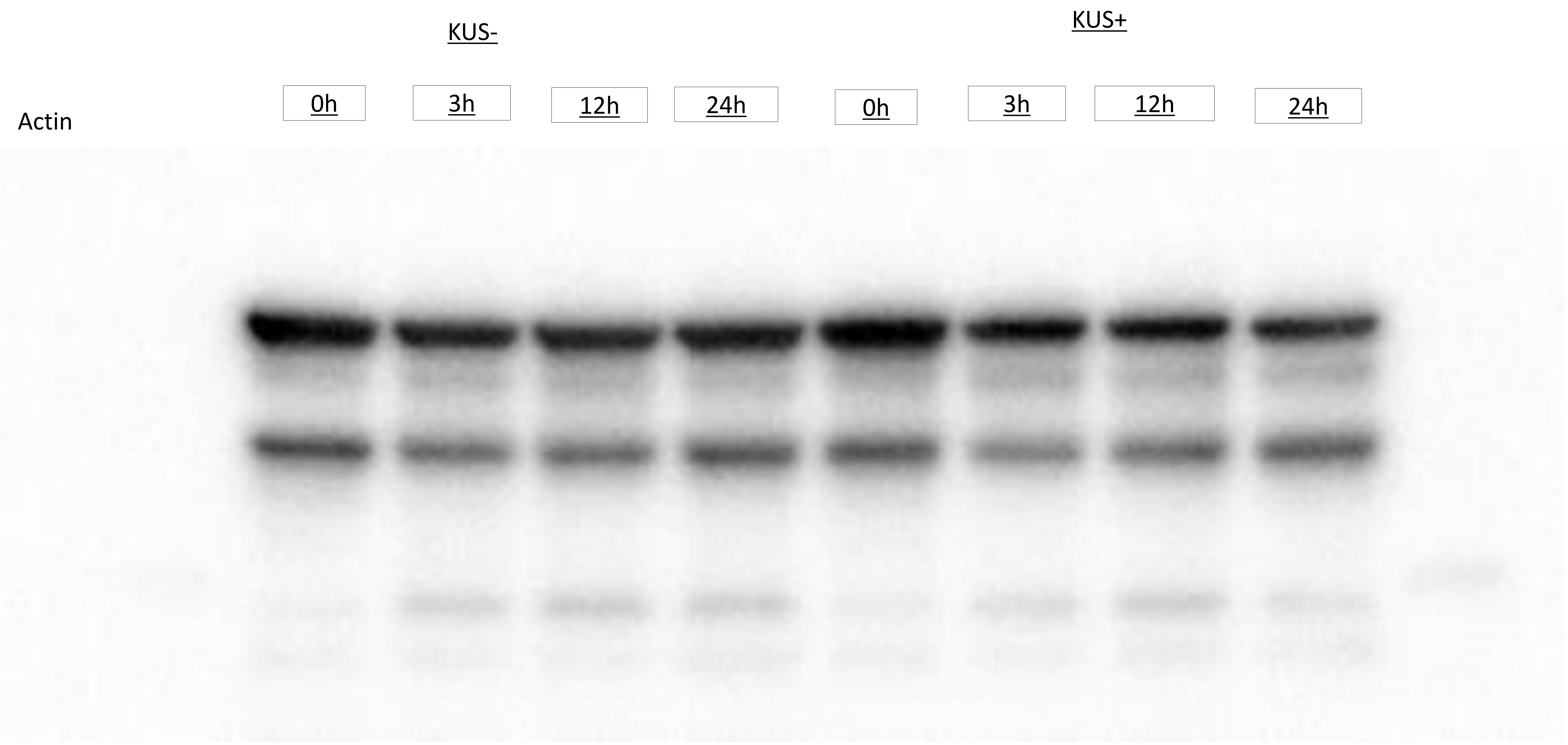

Full unedited gel for Figure 5g

Cleaved caspase-3

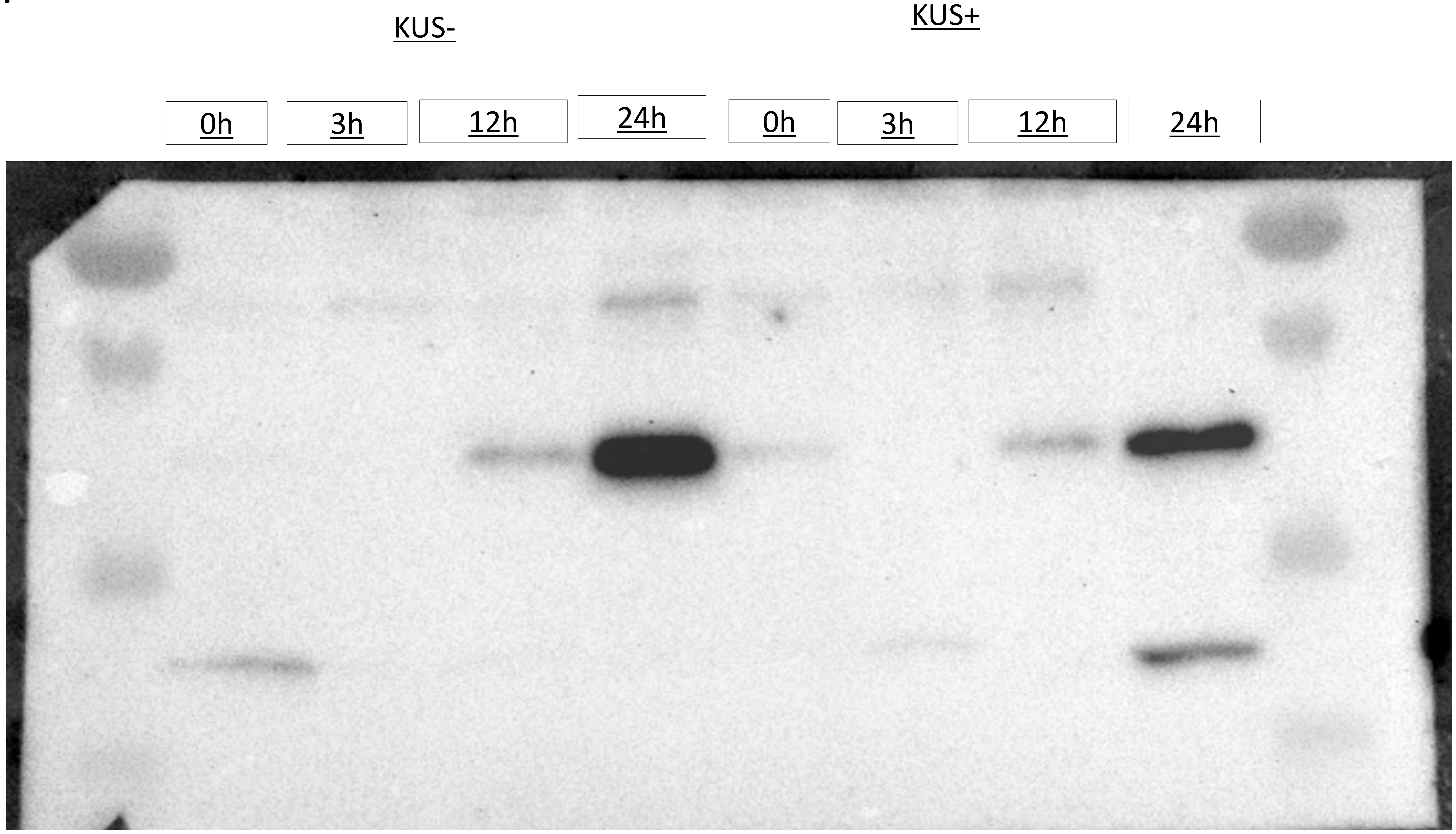

Full unedited gel for Figure 5g

KUS- KUS+

Actin

0h 3h 12h 24h 0h 3h 12h 24h

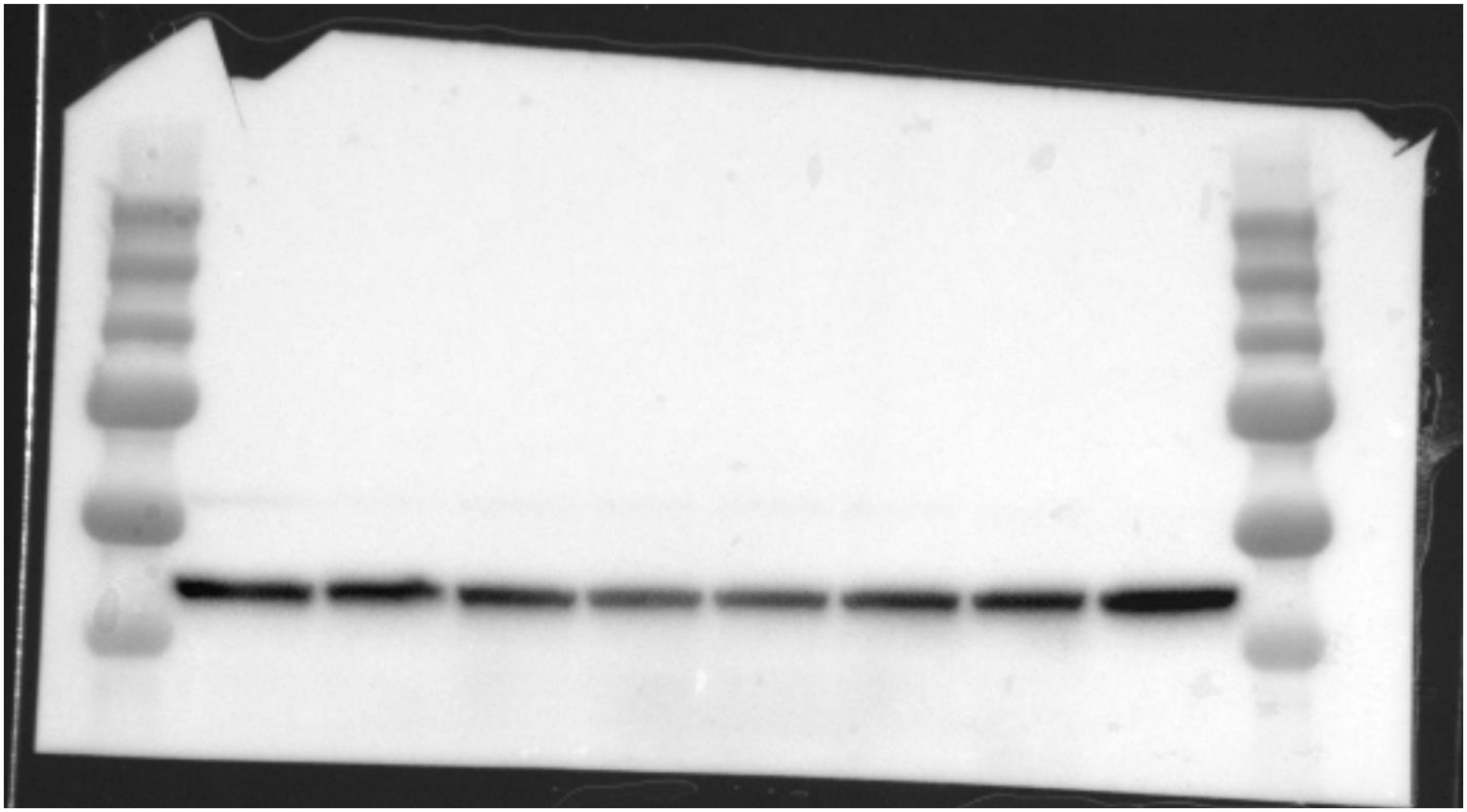

Supplement: Supplemental Figure 1 [file srep44873-s1.pdf]
